# Current status and trends of urinary incontinence research in recent 10 years based on VOSviewer visualized analysis: An observational study

**DOI:** 10.1097/MD.0000000000039526

**Published:** 2024-08-30

**Authors:** Lei Wang, Zhicheng Luo, Pengpeng Zhao, Yi Yu, Yang Chen, Fuxiang Lin, Zhanping Xu

**Affiliations:** a The Eighth Clinical Medical College, Guangzhou University of Traditional Chinese Medicine, Foshan, Guangdong, China; b Taishan Hospital of Traditional Chinese Medicine, Jiangmen, Guangdong, China; c Foshan Hospital of Traditional Chinese Medicine, Foshan, Guangdong, China.

**Keywords:** bibliometrics, urinary incontinence, visual analysis, VOSviewer, Web of Science

## Abstract

Urinary incontinence (UI) is prevalent and imposes significant social and economic burdens. This study reviews the literature on UI, assesses the current research status, and projects future trends. To visualize and analyze UI-related research, summarize and generalize the knowledge framework of the global UI field, and explore the current state of research and emerging trends, we conducted a comprehensive search of UI-related studies from 2014 to 2024 using the Web of Science Core Collection. Utilizing VOSviewer software, we mapped the scientific landscape and performed visual analytics on collaborative and co-citation networks, keyword co-occurrences, emergent clusters, and timeline mapping to elucidate the research dynamics. A total of 4326 publications were retrieved for further analysis. The United States ranked first in terms of the total number of publications, number of citations, and publication H-index. In terms of institutions, the University of California System had the most total citations and the highest H-index. Neurology and Urodynamics had the most articles published, the highest citation frequency, and the highest H-index. The author with the most citations and the highest average number of citations per article is Abrams P. The author with the highest H-index is Peyronnet B. Based on the keyword analysis, the articles were categorized into several main directions: epidemiological studies, diagnostic studies, treatment studies, female UI studies, and male UI studies. Epidemiology, treatment, and male UI are expected to continue as hot topics. This study demonstrates that UI research is more advanced in Europe and North America and that Neurourology and Urodynamics is the most influential journal in the field. In addition, epidemiology, treatment, and male UI will continue to be prominent topics. Our study contributes to a more comprehensive understanding of the current state of UI research and provides insights into future research directions in the field.

## 1. Introduction

Urinary incontinence (UI) is the involuntary leakage of urine, significantly impacting a patient’s quality of life.^[1]^ UI affects approximately 2 billion people worldwide, with 23% to 45% of the female population experiencing varying degrees of incontinence and about 7% suffering from significant symptoms.^[2]^ In men, UI is most commonly caused by prostate enlargement or damage from surgical or radiation treatment for prostate cancer.^[3]^ In contrast, female UI is typically associated with dysfunction of the bladder and pelvic floor muscles, often occurring during pregnancy, childbirth, or menopause.^[4]^ Female UI is primarily categorized into stress UI and urge UI. In stress UI, leakage occurs with physical exertion, while in urge UI, leakage is associated with a sudden, intense urge to urinate. Women exhibiting both symptoms are classified as having mixed incontinence.^[5,6]^ As a prevalent condition, UI severely diminishes health-related quality of life and imposes substantial personal and socioeconomic burdens. Despite its prevalence, UI is frequently under-diagnosed and under-treated.^[7,8]^

Over the past decade, numerous studies have extensively explored UI, contributing significantly to advancements in diagnosis and treatment. This study aims to provide a systematic assessment of UI-related research using bibliometric analysis methods. The study will use mathematical and statistical tools to perform quantitative and qualitative analyses of relevant academic papers and other publications, leading to detailed bibliometric analyses of authorial collaborations, thematic co-occurrences, co-citation of literature, and reference coupling.^[9,10]^ Bibliometric methods have indeed been widely employed in various areas of medical research.^[11–13]^

Our goal is to survey the current status and trends in global UI research, provide a comprehensive analysis of the field, identify research hotspots and emerging trends, determine cutting-edge directions, and offer valuable insights. We hope that our study will not only establish a systematic and comprehensive knowledge base for both experienced and novice researchers but also offer valuable references for scientific decision-making, research management within research organizations, and academic exploration.

## 2. Information and methods

### 2.1. Data collection

**Figure 1.**
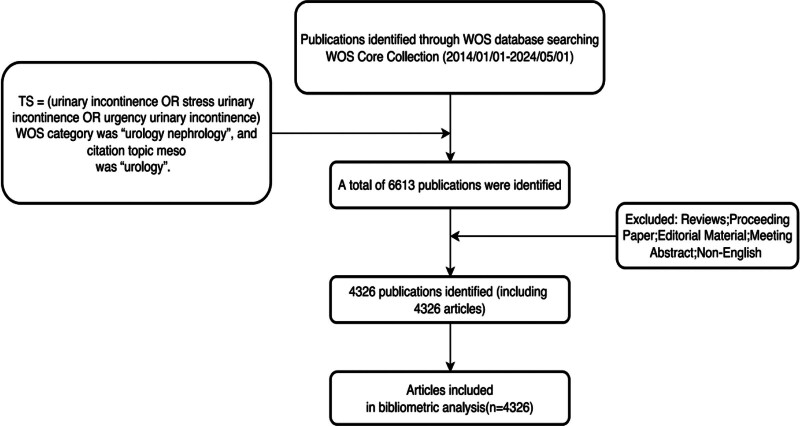
Detailed flowchart of search.

Data were collected from the Web of Science (WOS) Core Collection database, covering the period from January 1, 2014, to May 1, 2024. The data were independently evaluated by 2 researchers, Lei Wang and Peng-Peng Zhao. Any disagreements were resolved through consultation with experienced corresponding authors until a consensus was reached. The search strategy utilized the following terms: TS = (urinary incontinence OR stress urinary incontinence OR urgency urinary incontinence). The WOS category was “Urologic Nephrology” and the citation subject medium category was “Urology.” Only documents classified as “Article” were included to exclude non-English documents, reviews, patents, conference abstracts, books, and other irrelevant documents. After the initial search, results were manually screened, resulting in a total of 4326 relevant documents. Detailed information from each document was extracted for subsequent analysis, including title, keywords, author, country, affiliation, year of publication, references, and citations. Refer Figure 1 for details.

### 2.2. Bibliometric analysis and visualization

In this study, PRISM 9 software was used to analyze annual publications and visualize publication trends, while VOSviewer software was employed for bibliometric analysis and visualization. Using the WOS analysis tool, we collected data on the year of publication, total citation frequency, average citation frequency, H-index, and other basic characteristics of the papers. VOSviewer software version 1.6.19 (Leiden University, Leiden, The Netherlands) was used to construct and visualize literature networks based on co-authorship, co-occurrence, co-citation, and bibliographic coupling relationships.^[14–16]^ VOSviewer facilitates a better understanding and interpretation of different terms and clusters by creating network visualization maps, overlay visualization maps, and density visualization maps.

## 3. Results

### 3.1. Volume of literature published and growth trends

**Figure 2.**
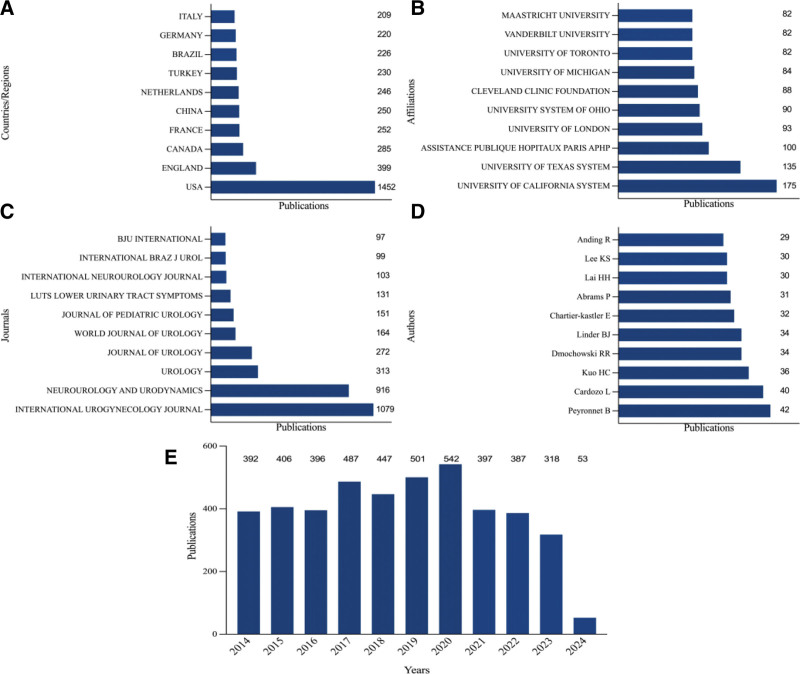
Number and trend of global UI research papers.

A total of 4326 publications were screened from WOS. Figure 2 presents the top 10 countries/regions, affiliations, journals, and authors in terms of the number of articles, as well as the growth trend of the literature over the past decade. The United States leads the field of UI research with 1452 publications, accounting for 33.564% of the global total. The United Kingdom follows with 399 publications, representing 9.223% of the total, and Canada ranks third with 285 publications, or 6.588% (see Fig. 2A). The UNIVERSITY OF CALIFORNIA SYSTEM leads all educational institutions with 175 publications, followed by the UNIVERSITY OF TEXAS SYSTEM with 135 publications, and ASSISTANCE PUBLIQUE HOPITAUX PARIS (AP-HP) with 100 publications. The top 10 universities with the highest number of publications are located in North America and Europe (see Fig. 2B). The top 10 journals with the highest number of published articles are presented in Figure 2C. The journal with the most articles was *INTERNATIONAL UROGYNECOLOGY JOURNAL* (1079), followed by *NEUROUROLOGY AND URODYNAMICS* (916) and *UROLOGY* (313). The author with the most publications was Peyronnet B (42 articles), followed by Cardozo L with 40 articles, and Kuo HC with 36 articles (see Fig. 2D). The number of articles published in this field has remained relatively stable, peaking at 542 in 2020, with a slight decline observed in the number of articles published after 2020 (see Fig. 2E).

### 3.2. Publication quality based on citations and the H-index

The total number of citations, the average number of citations per article, and the H-index of the top 10 countries/regions, affiliations, journals, and authors with the highest number of publications in the field of UI were analyzed and reported. The United States led with the highest total number of citations (20,381), while the United Kingdom had the highest average number of citations per article (19.96). Regarding the H-index, the United States had the highest value at 55, followed by the United Kingdom with 42 and Canada with 34. Concerning affiliations, the University of California System published the most articles, achieving the highest total number of citations (2524), the highest average number of citations per article (14.42), and the highest H-index (27). The most influential author, based on the highest number of citations (1079) and the highest average number of citations per article (34.81), was Abrams P. The author with the highest H-index (27) in this field was Peyronnet B (Table 1). Among journals, *Neurourology and Urodynamics* had the most cited articles (11,157) and the highest H-index (42), while *BJU International* had the highest average number of citations per article (26.57) (Table 2).

**Table 1:** Top 10 most published and cited counties/regions, organization, author in urinary incontinence studies.

		Publications (%)	Total citations	Average citations per item	H-index
Countries/Regions	USA	1452 (33.564)	20,381	17.48	55
	ENGLAND	399 (9.223)	7964	19.96	42
	CANADA	285 (6.588)	4615	16.19	34
	FRANCE	252 (5.825)	3515	13.95	30
	CHINA	250 (5.779)	2135	8.54	24
	NETHERLANDS	246 (5.687)	4483	18.22	33
	TURKEY	230 (5.317)	1977	8.6	21
	BRAZIL	226 (5.224)	2446	10.82	23
	GERMANY	220 (5.086)	4108	18.67	30
	ITALY	209 (4.831)	3530	16.89	30
Organization	UNIVERSITY OF CALIFORNIA SYSTEM	175 (4.045)	2524	14.42	27
	UNIVERSITY OF TEXAS SYSTEM	135 (3.121)	1958	14.5	21
	ASSISTANCE PUBLIQUE HOPITAUX PARIS APHP	100 (2.312)	1187	11.87	18
	UNIVERSITY OF LONDON	93 (2.15)	1760	18.92	23
	UNIVERSITY SYSTEM OF OHIO	90 (2.08)	1078	11.98	19
	CLEVELAND CLINIC FOUNDATION	88 (2.034)	1311	14.9	21
	UNIVERSITY OF MICHIGAN	84 (1.942)	1026	12.21	18
	UNIVERSITY OF TORONTO	82 (1.896)	1501	18.3	23
	VANDERBILT UNIVERSITY	82 (1.896)	1107	13.5	19
	MAASTRICHT UNIVERSITY	82 (1.896)	1616	19.71	21
Author	Peyronnet B	42 (0.971)	496	14.42	27
	Cardozo L	40 (0.925)	683	17.08	14
	Kuo HC	36 (0.832)	600	16.67	16
	Dmochowski RR	34 (0.786)	588	17.29	10
	Linder BJ	34 (0.786)	585	17.21	14
	Chartier-kastler E	32 (0.74)	419	13.09	11
	Abrams P	31 (0.717)	1079	34.81	15
	Lai HH	30 (0.693)	583	19.43	15
	Lee KS	30 (0.693)	439	14.63	11
	Anding R	29 (0.67)	371	12.79	12

**Table 2:** Top 10 most published and cited journals in the field of incontinence-related research.

Journal	Publications (%)	IF (2022)	JCR	Total citations	Average citations per article	H-index
INTERNATIONAL UROGYNECOLOGY JOURNAL	1079 (24.942)	1.8	Q4	10,004	9.27	35
NEUROUROLOGY AND URODYNAMICS	916 (21.174)	2	Q3	11,157	12.18	42
UROLOGY	313 (7.235)	2.1	Q3	3516	11.23	29
JOURNAL OF UROLOGY	272 (6.288)	6.6	Q1	6689	24.59	39
WORLD JOURNAL OF UROLOGY	164 (3.791)	3.4	Q2	1878	11.45	23
JOURNAL OF PEDIATRIC UROLOGY	151 (3.491)	2.0	Q3	1197	7.93	17
LUTS LOWER URINARY TRACT SYMPTOMS	131 (3.028)	1.3	Q4	812	6.2	13
INTERNATIONAL NEUROUROLOGY JOURNAL	103 (2.381)	2.3	Q2	791	7.68	15
INTERNATIONAL BRAZ J UROL	99 (2.288)	3.7	Q2	771	7.79	15
BJU INTERNATIONAL	97 (2.242)	4.5	Q1	2577	26.57	28

### 3.3. Author collaboration analysis

**Figure 3.**
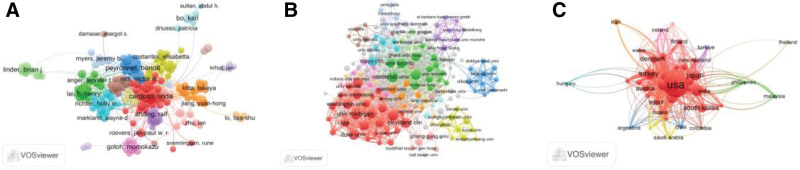
Analysis of coauthors of global UI studies. *Note*: (A–C) Web visualization maps display countries/regions, affiliations, and authors. Node size reflects the frequency of co-authorship, while connecting lines between nodes indicate established co-authorships. The color of each element represents its cluster, with different clusters distinguished by varying colors.

Coauthor analysis is used to analyze author collaboration based on co-published articles. Figure 3A shows that a total of 205 authors have published at least 10 articles. The author with the highest total link strength (TLS) is Anding, Ralf (164), followed by Bauer, Ricarda M (148), Kirkali, Ziya (148), Kretschmer, Alexander (147) and Queissert, Fabian (134), as detailed in Figure 3A. Figure 3B shows that a total of 240 organizations published at least 10 articles. The most TLS were at Duke University (285), Northwestern University (254), University of California, San Diego (245), University of Michigan (230), and Washington University (224). Figure 3C shows that a total of 47 countries/regions published at least 10 articles. The top 5 countries/regions with the most TLSs were the United States (861), the United Kingdom (733), the Netherlands (474), Canada (455), and Germany (405).

### 3.4. Keyword co-occurrence analysis

**Figure 4.**
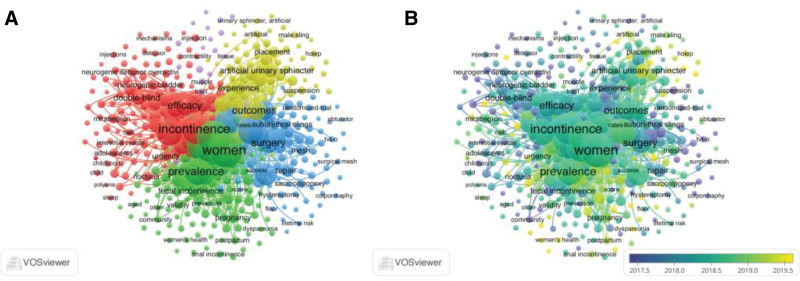
Co-occurrence analysis of global UI studies. *Note*: (A) Network visualization map of identified keywords. (B) Overlay visualization map of identified keywords to illustrate trends over time.

Co-occurrence analysis was used to reflect the strength of association between keywords, identify research hotspots, and determine the composition and paradigms of the disciplines represented by these keywords. This analysis also examined the development process and structural evolution of these fields both horizontally and vertically.^[17]^ Figure 4A shows that 411 keywords, each occurring at least 16 times, were categorized into 5 major research clusters based on the keyword clustering algorithm generated by VOSviewer. The same color indicates that the articles share a similar research scope. The red cluster (112) included keywords such as UI, overactive bladder syndrome, and efficacy. The green cluster (101) contained frequently occurring keywords such as female, stress incontinence, pelvic organ prolapse, and related terms. The blue cluster (89) included keywords such as surgery, repair, and prevalence. The yellow cluster (76) included keywords such as artificial urethral sphincter, male, and radical prostatectomy. The purple cluster (33) contained keywords such as muscle, tissue, and terminology. Overlaid visual maps were used to illustrate the evolution of keywords over time. The overall research trend shifted from a focus on diagnosis and female incontinence to treatment, epidemiology, and male incontinence (see Fig. 4B).

### 3.5. Co-citation analysis

**Figure 5.**
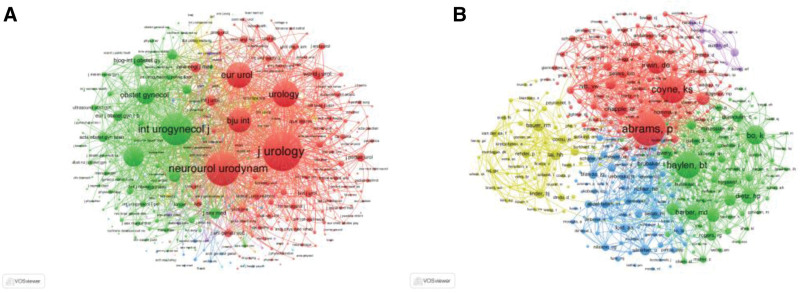
Common citation analysis of global UI studies. *Note*: (A and B) Network visualization maps for identified journals and authors. Node size reflects the frequency of citations. A line connecting 2 nodes indicates that the articles associated with these nodes were cited together in other literature.

Co-citation is the frequency with which 2 documents are cited by other documents at the same time.^[18]^ Figure 5A shows that a total of 414 journals had 20 co-citations. The journal with the highest TLS was the *Journal of Urology* (281, 102), followed by *Neurourology and Urodynamics* (226, 149), *International Urogynecology Journal* (212, 415), *Urology* (146, 939), and *European Urology* (143, 932). Figure 5B shows that a total of 371 authors had at least 40 co-citations. The top 5 authors with the highest TLS were Abrams P (12, 172), Coyne KS (8, 977), Haylen BT (8, 272), Irwin DE (5, 112), and Chapple CR (5, 090).

### 3.6. Bibliographic coupling analysis

**Figure 6.**
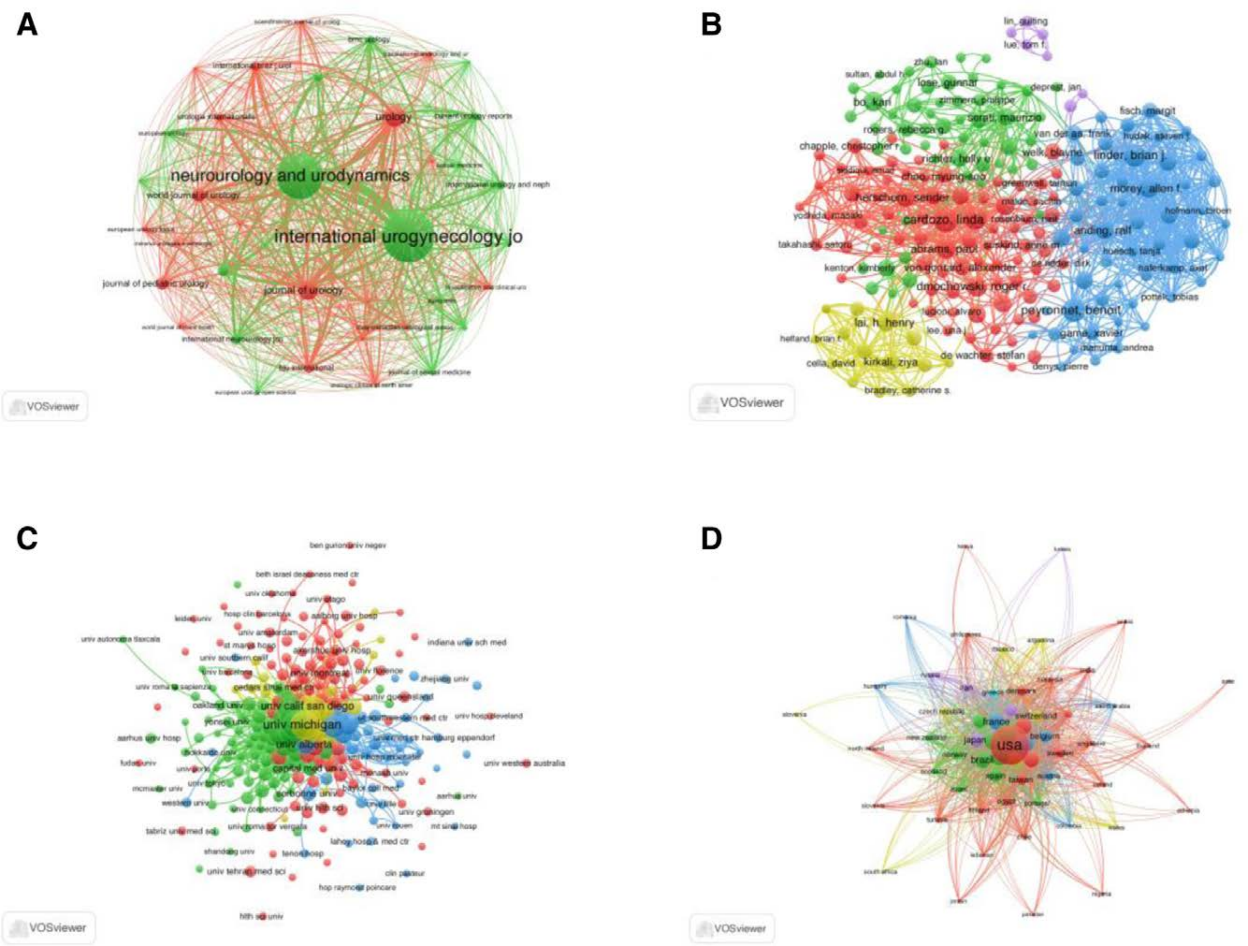
Bibliographic coupling analysis of global urinary incontinence research. *Note*: (A–D) Network visualization maps of identified journals, authors, affiliations, and countries/regions. Node size and line segments between nodes represent the degree of similarity among countries/regions, affiliations, journals, and authors.

Bibliographic coupling analysis was used to reflect the relationship and similarity between papers that cited a common third article.^[19]^ The results of the bibliographic coupling analysis for UI studies are shown in Figure 6. A total of 38 journals published at least 5 articles. The journal with the highest TLS was *Neurourology and Urodynamics* (302,843), followed by *International Urogynecology Journal* (282,455), *Urology* (106,675), *Journal* of *Urology* (102,101), and *World Journal of Urology* (63,935) (see Fig. 6A). A total of 205 authors published at least 10 articles. The author with the highest TLS was Bauer RM (26,501), followed by Anging R (25,247), Kretschmer A (24,347), Lai HH (23,951), and Serati M (22,200) (see Figure 6 B). A total of 245 affiliations with at least 10 publications were identified. The affiliations with the highest TLS were the University of Michigan (61,685), Washington University (60,378), Maastricht University (59,372), Duke University (59,362), and Northwestern University (51,263) (see Fig. 6C). Additionally, 59 countries/regions with at least 5 articles were identified. The top 5 countries/regions with the highest TLS were the USA (464,980), the UK (192,182), Canada (137,869), Germany (132,873), and France (119,052) (see Fig. 6D).

## 4. Discussion

Through a subject search, we screened 4326 articles related to UI published in journals indexed in the WOS from 2014 to 2024. These articles cover a broad range of UI research areas and provide valuable reference material for researchers and clinicians across various specialties, including medicine, nursing, and rehabilitation. This study aims to reveal and assess the progress and evolutionary trends in UI research across different subfields: such as epidemiology, diagnostic methods, treatment, and risk factors: over the past decade through an in-depth analysis of the literature.

Methodological and analytical tools are crucial for unraveling the pathogenesis of UI, assessing disease progression, and developing preventive and control measures and intervention strategies.^[20]^ This study focuses on the systematic analysis of UI literature through bibliometric and visualization methods. The current epicenter of UI research, including leading academic institutions, prominent journals, and active researchers, is predominantly located in Western countries, indicating a geographical concentration in research efforts. This concentration can be attributed to the higher awareness of incontinence diseases and the relatively frequent patient visits in developed Western countries. In contrast, despite China’s large population of incontinence patients, the number of related research papers published in China is limited, reflecting a lack of awareness, attention, and research investment in incontinence diseases. This highlights an urgent need to raise public awareness and increase research focus in China. According to our bibliometric analysis, the University of California System, University of Texas System, and Assistance Publique Hôpitaux de Paris (AP-HP) continue to dominate the current direction of incontinence research. Journals such as *International Urogynecology Journal*, *Neurourology and Urodynamics*, and *Urology* remain leading sources in incontinence research. The teams led by Peyronnet B, Cardozo L, and Kuo HC continue to be prominent leaders in this field. These journals and authors merit increased attention, as breakthroughs and discoveries in the field are more likely to emerge from their work. The research conducted by these teams is more likely to reflect the latest advances in UI research. The journal analysis suggests that communication and collaboration between international teams are increasing, and further efforts in this area are necessary to advance research in the field.

Using a bibliometric approach, Zhang et al^[21]^ provide a comprehensive overview of research in the field of stress urinary incontinence surgery from September 7, 2013, to September 7, 2023, highlighting key findings, research hotspots, and trends over the past decade. This study offers an objective and comprehensive overview of developments and trends in urinary incontinence research from 2014 to 2024. There is a slight decrease in the number of articles between 2021 and 2024, attributed to the COVID-19 pandemic. The overlaid visual map is color-coded by the year in which each keyword appears in the articles, illustrating progress over time. Analyzing the growth trends of keywords suggests that future research will increasingly focus on treatment, epidemiological characteristics, and male incontinence.

Treatment-related research aims to develop individualized treatment plans tailored to various types of incontinence, symptom severity, and frequency. These efforts seek to restore urinary control, reduce incontinence frequency, prevent complications, and ultimately improve patients’ quality of life. Epidemiological studies aim to raise public awareness of the physiological and psychological effects of UI, identify and avoid potential risk factors, and recognize protective factors to implement effective primary and secondary prevention measures. Male UI, a significant research topic, must be considered due to its impact on patients’ quality of life, even though its prevalence is approximately half that of female UI. Particularly noteworthy is the issue of UI following prostatectomy, with stress incontinence occurring in about 1% of patients after transurethral resection of the prostate (TURP) and ranging from 5% to 34% after radical prostatectomy.^[22]^ Although focused, these research areas provide a diverse perspective on UI research and encourage a deeper exploration and understanding of incontinence disorders.

Inevitably, there were some limitations in the design and execution of this study that need to be addressed. First, the literature screening was confined to papers published after 2014 and only included articles categorized under “urologic nephrology” in the WOS database. This restriction may have led to the exclusion of significant incontinence-related research that falls outside these parameters. Second, data extraction relied solely on the WOS Core Collection database, which might not fully capture the overall landscape of incontinence research. While the bibliometric analysis and visualization methods employed in this study offer advantages in terms of objectivity and comprehensiveness compared to other approaches, the analysis remains limited by the scope of the databases used. Important databases such as PubMed, Scopus, and Google Scholar were not included, potentially omitting valuable research contributions.

Additionally, some articles with high academic value were cited fewer times than expected, possibly reflecting a bias in the perception of their impact. Future research should consider incorporating a broader range of database resources to achieve more comprehensive and reliable results. By expanding the scope of data sources, subsequent studies can provide a more holistic view of incontinence research and address the limitations encountered in this study.

## 5. Conclusions

Using bibliometric methods, this study provides a systematic analysis of global academic publications on UI. It presents a comprehensive assessment of the current research status in the field and projects future research trajectories. Currently, the primary hotspots of UI research are concentrated in Europe and North America. Among the numerous academic journals, the 3 with the highest number of incontinence-related publications are the *International Urogynecology Journal, Neurourology and Urodynamics,* and *Urology*. It is anticipated that epidemiologic studies and treatment strategies for UI, particularly male incontinence, will continue to be a prominent area of research. Specifically, bibliometric and visualization studies on UI are invaluable for guiding and inspiring future research directions, as they highlight emerging problems and key topics in the field.

## Author contributions

**Conceptualization:** Lei Wang, Pengpeng Zhao.

**Data curation:** Lei Wang, Pengpeng Zhao, Yi Yu.

**Formal analysis:** Lei Wang, Pengpeng Zhao, Yi Yu.

**Project administration:** Zhanping Xu.

**Resources:** Zhanping Xu.

**Software:** Lei Wang, Zhicheng Luo, Yang Chen, Fuxiang Lin.

**Supervision:** Lei Wang, Yi Yu, Yang Chen, Fuxiang Lin.

**Validation:** Lei Wang, Pengpeng Zhao.

**Visualization:** Zhanping Xu.

**Writing – original draft:** Lei Wang.

**Writing – review & editing:** Lei Wang, Zhicheng Luo.
